# Vaccine-linked chemotherapy with a low dose of benznidazole plus a bivalent recombinant protein vaccine prevents the development of cardiac fibrosis caused by *Trypanosoma cruzi* in chronically-infected BALB/c mice

**DOI:** 10.1371/journal.pntd.0010258

**Published:** 2022-09-12

**Authors:** Victor Manuel Dzul-Huchim, Maria Jesus Ramirez-Sierra, Pedro Pablo Martinez-Vega, Miguel Enrique Rosado-Vallado, Victor Ermilo Arana-Argaez, Jaime Ortega-Lopez, Fabian Gusovsky, Eric Dumonteil, Julio Vladimir Cruz-Chan, Peter Hotez, María Elena Bottazzi, Liliana Estefania Villanueva-Lizama

**Affiliations:** 1 Laboratorio de Parasitología, Centro de Investigaciones Regionales Dr. Hideyo Noguchi, Universidad Autónoma de Yucatán, Mérida, Yucatán, México; 2 Laboratorio de Farmacología, Facultad de Química, Universidad Autónoma de Yucatán, Mérida, Yucatán, México; 3 Departamento de Biotecnología y Bioingeniería, Centro de Investigación y Estudios Avanzados del Instituto Politécnico Nacional, Ciudad de México, México; 4 Eisai, Inc., Eisai Inc, Andover, Massachusetts, United States of America; 5 Department of Tropical Medicine, School of Public Health and Tropical Medicine, and Vector-Borne and Infectious Disease Research Center, Tulane University, New Orleans, Los Angeles, United States of America; 6 Texas Children’s Center for Vaccine Development, Departments of Pediatrics and Molecular Virology & Microbiology, Baylor College of Medicine, Houston, Texas, United States of America; 7 Department of Biology, Baylor University, Waco, Texas, United States of America; INGEBI, ARGENTINA

## Abstract

**Background:**

Chagas disease (CD) is caused by *Trypanosoma cruzi* and affects 6–7 million people worldwide. Approximately 30% of chronic patients develop chronic chagasic cardiomyopathy (CCC) after decades. Benznidazole (BNZ), one of the first-line chemotherapy used for CD, induces toxicity and fails to halt the progression of CCC in chronic patients. The recombinant parasite-derived antigens, including Tc24, Tc24-C4, TSA-1, and TSA-1-C4 with Toll-like receptor 4 (TLR-4) agonist-adjuvants reduce cardiac parasite burdens, heart inflammation, and fibrosis, leading us to envision their use as immunotherapy together with BNZ. Given genetic immunization (DNA vaccines) encoding Tc24 and TSA-1 induce protective immunity in mice and dogs, we propose that immunization with the corresponding recombinant proteins offers an alternative and feasible strategy to develop these antigens as a bivalent human vaccine. We hypothesized that a low dose of BNZ in combination with a therapeutic vaccine (TSA-1-C4 and Tc24-C4 antigens formulated with a synthetic TLR-4 agonist-adjuvant, E6020-SE) given during early chronic infection, could prevent cardiac disease progression and provide antigen-specific T cell immunity.

**Methodology/ Principal findings:**

We evaluated the therapeutic vaccine candidate plus BNZ (25 mg/kg/day/7 days) given on days 72 and 79 post-infection (p.i) (early chronic phase). Fibrosis, inflammation, and parasite burden were quantified in heart tissue at day 200 p.i. (late chronic phase). Further, spleen cells were collected to evaluate antigen-specific CD4^+^ and CD8^+^ T cell immune response, using flow cytometry. We found that vaccine-linked BNZ treated mice had lower cardiac fibrosis compared to the infected untreated control group. Moreover, cells from mice that received the immunotherapy had higher stimulation index of antigen-specific CD8^+^Perforin^+^ T cells as well as antigen-specific central memory T cells compared to the infected untreated control.

**Conclusions:**

Our results suggest that the bivalent immunotherapy together with BNZ treatment given during early chronic infection protects BALB/c mice against cardiac fibrosis progression and activates a strong CD8^+^ T cell response by *in vitro* restimulation, evidencing the induction of a long-lasting *T*. *cruzi*-immunity.

## Author summary

Chagas disease (CD) is a neglected tropical disease caused by the parasite *Trypanosoma cruzi*, transmitted through contact with infected feces of vectors bugs. CD can induce cardiac abnormalities including the development of fibrosis and eventually death. Benznidazole (BNZ) is the first-line drug approved against CD, however, its toxicity and lack of efficacy in the chronic phase have limited its use. Previous studies have demonstrated the feasibility of reducing doses of BNZ given in combination with therapeutic vaccines during the acute phase of CD, which increases its tolerability and reduces adverse side effects. Considering that patients are often diagnosed until more advanced stages of the disease, its necessary to evaluate therapies given in the chronic phase of CD. In this study, we evaluated a vaccine formulated with the recombinant *T*. *cruzi*-antigens TSA-1-C4 and Tc24-C4 and the adjuvant E6020-SE in combination with a low dose of BNZ given during the chronic phase of *T*. *cruzi*-infection using a murine model. The authors found that the combination therapy protects mice against cardiac fibrosis progression, allows the activation of a CD8^+^ T cell response, and induces a prolonged memory response against *T*. *cruzi*. This study supports the development of the vaccine-linked chemotherapy approach to prevent *T*. *cruzi* chronic infection.

## Introduction

Chagas disease (CD) is caused by the protozoan parasite *Trypanosoma cruzi* (*T*. *cruzi*) transmitted mainly through contact with infected feces of hematophagous triatomine bugs. CD affects approximately 6.5 million people worldwide and is a major public health problem in Latin America [1]. Moreover, CD is emerging in non-endemic regions due to human migration, political and socioeconomic instability, climate change and other factors [2]. CD has two major clinical stages. The first is the acute infection and is characterized by high levels of parasites in peripheral blood; individuals are mostly asymptomatic but can present non-specific febrile illness, which typically resolves within 4–8 weeks [3,4]. Otherwise, the disease progresses into a chronic phase, where approximately 20–30% of individuals develop chronic chagasic cardiomyopathy (CCC) years to decades after the initial infection, and some develop pathologies such as megaesophagus and megacolon [5,6]. The pathogenesis of CCC is due to *T*. *cruzi* persistence in the heart that drives chronic inflammation and fibrosis leading to abnormalities of the conduction system [4,7,8]. Benznidazole (BNZ), is one of the first-line chemotherapies used for CD treatment, however, it is associated with toxic side effects, has a poor efficacy in patients with chronic infection and requires long treatment, increasing the risk of drug resistance [9]. Furthermore, therapy with BNZ does not reduce cardiac clinical deterioration through 5 years of follow-up in CCC patients [10]. Conversely, other studies have reported that BNZ treatment is associated with a reduction in heart disease progression, suggesting that more trials focused on BNZ should be performed [11]. Given the difficulties of human trials, the use of murine models has an important role in CD research. Previous studies have showed that, similar to humans, when *T*. *cruzi*-infected mice receive BNZ at standard dose (100mg/kg) in acute phase, the development of pathologies are hampered, however, when treatment is given in chronic phase, the effectiveness varies, and in some cases, cardiac fibrosis develops [12,13]. Nonetheless, some variations can be presented according to variables such as mouse model, parasite strain, drug dose administered, and treatment scheme [14,15].

The development and use of therapeutic vaccines represent an attractive alternative approach against *T*. *cruzi* infection. BALB/c mice have been widely used as experimental model in the development of therapies against CD given that they imitate aspects of human pathogenesis (immunological, pathological, and physiological) [16–18]. In the last years, several therapeutic vaccine candidates have been evaluated exploring a diversity of delivery systems (plasmids, adenoviruses, peptides and recombinant proteins) and adjuvants [19–23]. Overall, in animal models of *T*. *cruzi* experimental infection, either T helper (Th)-1 or Th1/Th2 balanced and Th-17 immune responses are required to achieve parasite control [24–26] with evidence for the importance of IFNγ and CD8^+^ T cells [27–32]. Furthermore, a vaccine candidate that can induce lasting memory-response is expected to prevent infection. According to the model proposed by Lanzavecchia and Sallusto [33], based on the expression of receptors required for lymph node homing, memory T cells are classified as effector memory (T_EM_), which can migrate to inflamed peripheral tissues and display immediate effector function, or central memory (T_CM_) which remain in secondary lymphoid organs, have little or no effector function, but readily proliferate and differentiate to effector cells. Therefore, therapeutic vaccination strategies have focused on promoting antigen-specific CD4^+^ and CD8^+^ memory T cells during the chronic phase of *T*. *cruzi-*infection [34–37], which is also when most Chagasic patients seek treatment.

Our program has been examining the effects of two major recombinant protein antigens, together with Toll-like receptor 4 (TLR-4) agonist adjuvants. These antigens include a flagellar calcium-binding protein, Tc24, or a genetically re-engineered Tc24 antigen with cysteine modifications to prevent aggregation, known as Tc24-C4 [38,39], and a trypomastigote surface antigen known as TSA-1 [40] or genetically re-engineered TSA-1-C4. Hence, both proteins are being produced under current good manufacturing practices (cGMP) as potential vaccines. Studies performed in acute *T*. *cruzi*-infection in mice indicate that both Tc24 and TSA-1 recombinant proteins drive a Th1 or balanced Th1/Th2 immunity in achieving therapeutic effects, with an emphasis on their role in reducing parasite persistence in the heart, and the associated fibrosis and inflammation [22–24,41,42]. In addition, the synthetic phospholipid dimer, E6020 (an agonist of TLR-4) alone or formulated with *T*. *cruzi* recombinant antigens in a squalene emulsion demonstrated its ability to decrease cardiac parasite burden and increase survival in BALB/c mice with acute *T*. *cruzi* infection [23,38,40,42–44]. Moreover, a recent study also has confirmed the immunogenicity of *T*. *cruzi* vaccines formulated with the E6020-SE adjuvant in *Rhesus* macaques [45]. While most of the studies in mice have focused on immunizations using a single recombinant protein antigen, our earlier work using plasmid DNA immunization evaluating bivalent vaccines with both TSA-1 and Tc24 [46], demonstrated that there is a beneficial effect to use bivalent vaccines during acute infection.

On the other hand, BNZ dosage and treatment regimens have been controversial in recent years. To assess the feasibility of minimizing the toxic side effects, several trials have been performed to test low doses of BNZ given alone or in combination with other drugs or antigens [47–50]. Accordingly, combined regimens improve the trypanocide activity and attenuate the BNZ toxicity, thus, these studies support the evaluation of an immunotherapy based on a reduced low-dose of BNZ linked to a vaccine formulated with the recombinant proteins TSA-1-C4 and Tc24-C4. Recently, studies have demonstrated the efficacy of the recombinant Tc24-C4 antigen in combination with a low dose of BNZ administered during *T*. *cruzi* acute infection in a murine model resulting in increased antigen-specific CD8^+^ and IFNγ-producing CD4^+^ T cells populations, as well as in increased cytokines related to Th17 immune responses [44]. Although the bivalent vaccine has showed immunogenicity and protection in acute murine models, it needs to be evaluated in pre-clinical models of *T*. *cruzi* chronic infection.

Our group previously performed a pilot study in order to evaluate the progression of *T*. *cruzi* chronic infection in BALB/c mice based on the parasitemia and cardiac clinical manifestations using electrocardiograms (taken every 35 days) and echocardiograms (at 210 dpi). According to the data, parasitemia was absent at day 50 p.i suggesting the beginning of the chronic phase. Cardiac clinical manifestations such as reduction in cardiac frequency and increase in the ejection fraction were present at day 140 p.i. allowing us to differentiate between the chronic asymptomatic and symptomatic stage. Those results suggested that days 72 and 200 p.i. are representative time points of the early and late phases of chronic infection respectively. In this study, we evaluated an immunotherapy administrated during the early chronic phase of experimental *T*. *cruzi* infection. We hypothesized that a low dose BNZ treatment in combination with a therapeutic vaccine (TSA-1-C4 and Tc24-C4 recombinant antigens in a formulation with a synthetic TLR-4 agonist-adjuvant, E6020-SE) given during early chronic infection could prevent cardiac disease progression and provide antigen-specific T cell immunity. This study could support the use of the vaccine-linked chemotherapy approach given in people during chronic infection, preventing or delaying the development of severe manifestations in prolonged stages of CD.

## Materials and methods

### Ethics statements

All studies were approved by the institutional bioethics committee of the “Centro de Investigaciones Regionales Dr. Hideyo Noguchi”, Universidad Autónoma de Yucatán (Reference #CEI-08-2019) and were performed in strict compliance with NOM-062-ZOO-1999.

### Proteins, adjuvant and benznidazole

The recombinant TSA-1-C4 and Tc24-C4 antigens were obtained from the Centro de Investigación y Estudios Avanzados (CINVESTAV) of the Instituto Politécnico Nacional (IPN), Mexico. Each TSA-1-C4 or Tc24-C4 coding sequence was cloned into a pET41a+ *E*. *coli* expression vector. The resulting plasmid DNA was transformed into BL21 (DE3) cells induced with isopropyl-beta-D-1-thiogalactoside (IPTG) for protein expression. Recombinant proteins were purified by ion exchange (IEX) and size exclusion chromatography (SEC) [38,40]. The integrity and size of each recombinant protein were analyzed by SDS-PAGE electrophoresis (**S1 Fig**). The recombinant proteins were formulated with the adjuvant E6020 in a stable squalene emulsion (SE). E6020 Toll-like receptor 4 agonist was acquired through Eisai, Inc [51]. Benznidazole (N-Benzyl-2-nitro-1H-imidazole-1-acetamide) was obtained through Sigma Aldrich; for its use, it was solubilized in 5% dimethyl sulfoxide (DMSO)-95% deionized water [42,44].

### Mice and parasites

Female BALB/c mice (BALB/cAnNHsd) were obtained at 3–4 weeks old from ENVIGO-Mexico. Animals were housed in groups of 5 per cage, received *ad libitum* food and water and a 12-h light/dark cycle. Mice were acclimated for two weeks before starting the study. *T*. *cruzi* H1 parasites, originally isolated from a human case in Yucatan, Mexico were maintained by serial passage in BALB/c mice every 25 to 28 days and used for infections as previously described [52].

### Macrophages cell line

RAW 264.7 cell line was acquired from American type culture collection (ATCC TIB-71). Cells were cultured in DMEM medium (Gibco) supplemented with 10% fetal bovine serum (FBS, Gibco) and 1% penicillin/streptomycin (Gibco), in an atmosphere of 5% CO_2_ and 95% humidity at 37°C. Cells were passaged in T-75 culture flasks (Corning) after reaching 80% confluence and were detached using 0.25% trypsin (Corning).

### Experimental infection and therapeutic treatment

A total of 70 mice were infected with 500 trypomastigotes of *T*. *cruzi* (H1 strain) by intraperitoneal injection. In order to confirm the infection, parasitemia was measured in Neubauer chamber by examination of fresh blood collected from the mouse tail on day 27 post-infection. Survival was monitored up to day 200 post-infection (p.i). On day 72 p.i. (early chronic phase) surviving mice were randomly divided into groups of 8 individuals and the therapeutic vaccine was injected intramuscularly. A vaccine boost with the same formulation was administrated one week after (day 79 p.i.). Each vaccine dose consisted of 12.5 μg of recombinant TSA-1-C4, 12.5 μg of recombinant Tc24-C4 and 5 μg of E6020-SE [38,42]. From day 72 to 79 p.i., mice were given daily 25 mg/kg BNZ by oral gavage, which corresponds to a 4-fold reduction in the conventional regimen of BNZ treatment. Additional groups of mice received the therapeutic vaccine alone (12.5 μg of TSA-1-C4, 12.5 μg of Tc24-C4 and 5 μg of E6020-SE), low dose BNZ alone (25 mg/kg), E6020-SE alone (5 μg) or saline solution as control. Groups that did not receive BNZ were given the vehicle solution (5% DMSO in 95% deionized water) by oral gavage. One additional control group with four non-infected mice was also included. At day 200 p.i., all mice (35 weeks old) were euthanized using ketamine/xylazine-induced deep anaesthesia followed by cervical dislocation, and spleens and hearts were collected for further analysis.

### Quantification of parasite burden

Total DNA was isolated from cardiac tissue using the Kit Wizard Genomic DNA purification (Promega Madison WI). Each sample was quantified employing a BioSpec-nano spectrophotometer system (SHIMADZU) and adjusted to 50 ng of DNA from cardiac tissue, then quantitative real-time PCR (qPCR) was performed in an Illumina Eco thermocycler using SYBR Green Master Mix 1X and primers TCZ-F 5’-GCTCTTGCCCACAMGGGTGC-3’ and TCZ-R 5’-CCAAGCAGCGGATAGTTCAGG-3, which amplify a 188 pb product from the satellite region of *T*. *cruzi* DNA [53,54]. Cardiac parasite burdens were calculated based on a standard curve and expressed as parasite equivalents per 50 ng *T*. *cruzi*-DNA [55].

### Cardiac fibrosis and inflammation

For histopathological analysis, cardiac tissue from euthanized mice was fixed in 10% neutral buffered formalin solution, embedded in paraffin, cut into 5 μm sections, and stained with either Masson’s trichrome or haematoxylin and eosin (H&E) for fibrosis or cardiac inflammation measurement, respectively. To assess cardiac fibrosis or cardiac inflammation, five to nine representative pictures from each slide stained were acquired at 10X (for fibrosis) or 20X (for inflammation) magnification using an OLYMPUS microscope (CX23) adapted with a digital camera MiniVID P/N TP605100 (LW Scientific). Image analysis was performed using ImageJ software version 2.0.0/1.52p. To quantify cardiac fibrosis, pixels corresponding to collagen (blue colored) were quantified from the myocardium section of each animal tissue ([23,44,56]. For inflammatory infiltrate cells, the number of pixels corresponding to total nuclei was quantified from the myocardium section to estimate the number of inflammatory infiltrate cells per mm^2^ [56,57]. Data is presented as cardiac fibrosis percentage area or cardiac inflammatory cells per mm^2^, respectively.

### Preparation of spleen mononuclear cells

Spleens were mechanically dissociated by being pressed through a 100 μm pore-size cell strainer. Splenocytes were rinsed with RPMI medium (Gibco) supplemented with 10% FBS and 1% penicillin-streptomycin (RPMIc) and pelleted by centrifugation for 5 min at 400 x *g* at room temperature. The supernatant was decanted, and the splenocyte pellet was resuspended in balanced salt solution buffer (BSS) pH 7.4. Afterwards, the splenocyte suspension was mixed with Ficoll-histopaque (GE Healthcare BIO-Sciences) solution in 3:4 proportion and centrifuged at 400 x *g* for 40 min. The mononuclear cell layer was collected and washed twice with BSS buffer. The cell pellet was resuspended in RPMIc medium, cell viability was assessed by Trypan blue exclusion test and cell numbers were determined in a Neubauer chamber.

### Intracellular cytokine and memory T cell immune phenotyping

A total of 5x10^5^ mononuclear cells were co-cultivated with RAW 264.7 macrophages previously stimulated with TSA-1-C4+Tc24-C4 (25 μg/mL final concentration) in 10:1 proportion. Co-cultures were incubated in 5% CO_2_ and 95% humidity at 37°C during 20 h for intracellular cytokine production or 96 h for memory immune-phenotyping assays. To evaluate intracellular cytokine production, brefeldin A (BD biosciences) was added to co-culture for the last 4 hours of incubation. Re-stimulated cells were collected and washed twice with PBS+BSA 0.01%, then, cells were stained with anti-CD3 Alexa-647 (BD biosciences), anti-CD4 PE-Cy7 (BD biosciences) and anti-CD8 PERCP-Cy5.5 (BD biosciences) [40] or anti-CD3 PE-Cy7 (BD biosciences), anti-CD4 APC (BD biosciences), anti-CD8 BB515 (BD biosciences), anti-CD44 PE (BD biosciences) and anti-CD62L BV510 (BD biosciences) for memory immune-phenotyping assay. For intracellular cytokine production, cells were fixed with Cytofix/Cytoperm (BD biosciences), and permeabilized according to the manufacturer’s instructions. Permeabilized cells were stained with anti-IFNγ (BD biosciences) and anti-Perforin (INVITROGEN). Cells were resuspended in FACS buffer and acquired on a BD FACSVerse flow cytometer. At least 50,000 total events in the mononuclear cell gate were obtained using FACSuite software version 1.0.5. Data were analyzed in FlowJo software version 10.0.7r2. The stimulation index was calculated with the frequency of cells (stimulated with TSA-1-C4+Tc24-C4) and the frequency of non-stimulated cells (RPMIc alone). For intracellular analysis, the stimulation index was measured with the median fluorescent intensity (MFI) of stimulated cells and the MFI of non-stimulated cells. A stimulation index > 1, indicates the presence of antigen-specific cells. Flow cytometry gating strategies for IFNγ and perforin expression or memory responses are presented in **S2** and **S3 Figs.**

### Statistical analysis

All tests were run in GraphPad Prism software version 6.0.c. Data were analyzed by one-way ANOVA or Kruskal–Wallis tests for multiple groups, depending on its distribution followed by Tukey or Dunn’s *post hoc* test. When only two comparison groups were analysed, Student´s t-test or Mann–Whitney U-test was performed depending on data distribution. Differences between groups were considered statistically significant when *P*-value was less than 0.05.

## Results

### Acute infection

**Fig 1:**
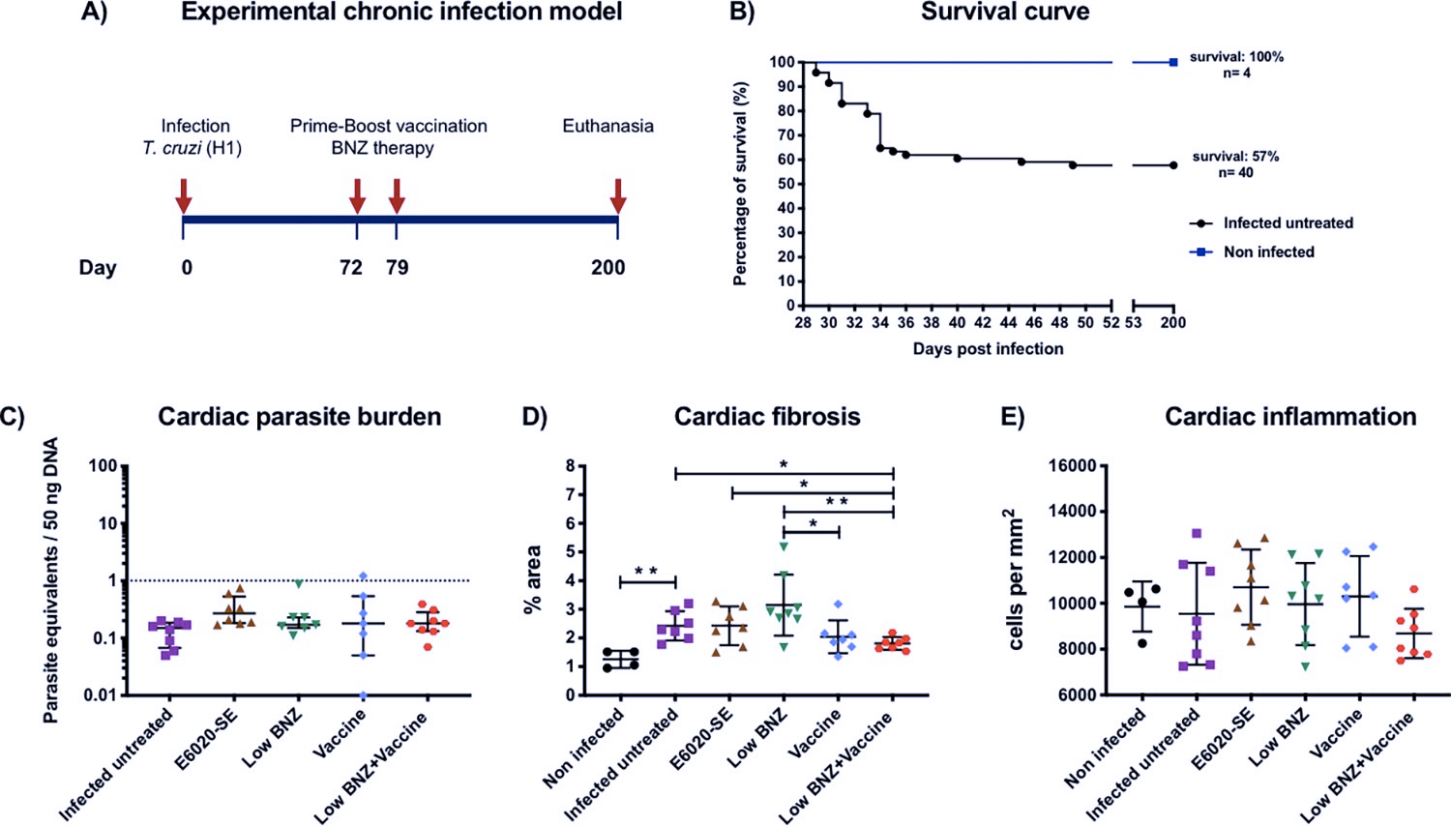
Protective effect of the vaccine-linked chemotherapy. **(A)** Timeline for experimental infection. A total of 70 BALB/c mice were infected with 500 trypomastigotes of *T*. *cruzi* (H1 strain) by intraperitoneal injection. Four mice were included as non-infected control group and received only saline solution. At day 200 p.i mice were euthanized, and tissues samples were collected. **(B)** Survival curve. Survival was monitored during 200 days post-infection. (**C**) Cardiac parasite burden was quantified by quantitative real-time PCR. The dotted line represents the cut-off for the limit of detectable quantification (LOQ) based on serially diluted *T*. *cruzi*-enriched cardiac tissue DNA (1 parasite equivalents per 50 ng of DNA). Each point represents an individual mouse; horizontal lines denote median ± interquartile ranges values; significance calculated by Kruskal-Wallis test with Dunn’s correction for multiple comparisons. (**D**) Percentage fibrosis area for all experimental groups. The cardiac fibrosis was quantified from representative images of Masson´s trichrome-stained tissue sections using Image J software. Each point represents an individual mouse, horizontal lines denote means ± SD; significance between two groups was calculated by Student´s t-test. (**E**) Infiltrate cells/mm^2^ for all experimental groups. Cardiac inflammation was quantified from representative images of H&E-stained tissue sections using Image J software. Each point represents an individual mouse, horizontal lines denote means ± SD; significance was calculated by ANOVA test. Significance is indicated as follows *, *P*≤0.05; **, *P*≤0.01.

A total of 70 mice were infected with 500 blood trypomastigotes, as described above. The timeline for experimental *T*. *cruzi*-infection is showed in **Fig 1A**. All infected mice were confirmed positive to *T*. *cruzi* by parasitemia measurement at day 27 p.i. (**S1 Table**). Mortality began at day 29 p.i. (peak of parasitemia) and continued progressively for three weeks (**Fig 1B**). As expected, by day 49 p.i. survival was reduced to 57% in infected mice compared to non-infected control group (100% survival). Survived mice (n = 40) were randomly divided in groups of 8 individuals for the immunotherapy experiment.

### Vaccine-linked chemotherapy administered during the early chronic infection prevents cardiac fibrosis caused by *T*. *cruzi*

To evaluate the therapeutic efficacy of the vaccine-linked treatment, we measured cardiac *T*. *cruzi*-parasite burden, fibrosis, and inflammation. At day 200 p.i. *T*. *cruzi* cardiac parasite burden from mice treated and untreated was below the limit of detection of our qPCR test (<1 parasite per 50 ng of cardiac tissue) (**Fig 1C**). There were no differences comparing all experimental groups (Kruskal-Wallis, *P* = 0.125). These data suggest that the methodology used has limitations in determining the therapeutic efficacy upon the burden parasite of the formulation during the late chronic phase of infection in BALB/c mice.

**Fig 2:**
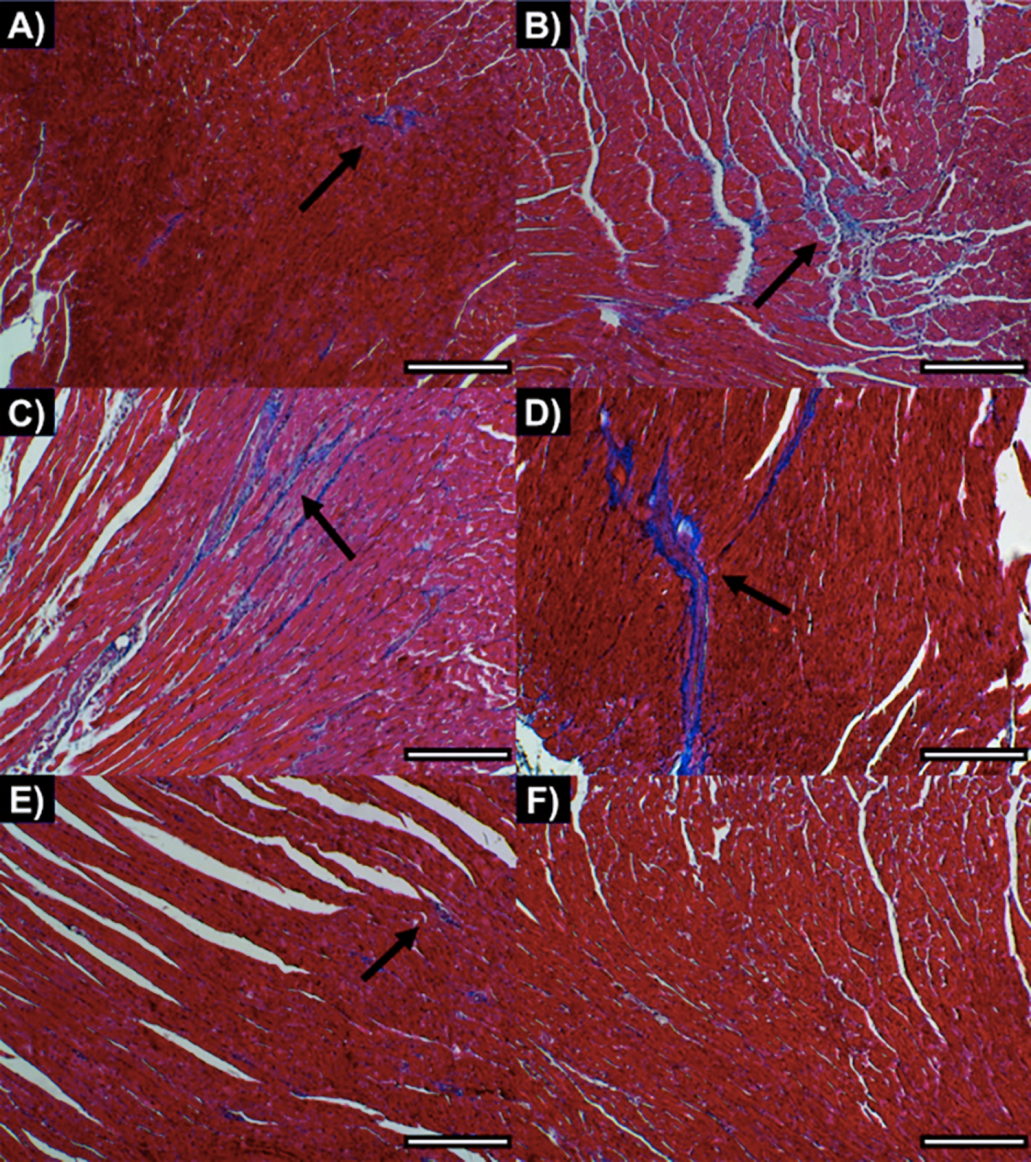
Representative images of Masson´s trichrome stained-cardiac tissue from (**A**) non-infected, (**B**) infected untreated, (**C**) E6020-SE, (**D**) low BNZ, (**E**) vaccine alone and (**F**) low BNZ plus vaccine experimental groups. Five-nine representative photographs were taken from the myocardial region and were acquired at 10X magnification using a microscope adapted with a digital camera. Cell cytoplasm appears in red and collagen fibbers in blue. Black arrows show fibbers of collagen. Scale bar corresponds to 10μm.

On the other hand, we evaluated cardiac fibrosis in heart tissue sections collected from *T*. *cruzi*-infected mice at day 200 p.i. Representative images of Masson´s trichrome stained-cardiac tissue from each experimental group are shown in **Fig 2A–2F**. As we observed in **Fig 1D**, there was a significantly higher percentage of cardiac fibrosis in infected untreated mice (2.426 ± 0.51) compared to non-infected mice (1.259 ± 0.299) (Student´s t-test, *P =* 0.003). Thus, mice infected with *T*. *cruzi* developed cardiac fibrosis as a consequence of chronic infection. Also, we found significant differences comparing the combination of low BNZ + vaccine (1.811 ± 0.223) with infected untreated mice (Student´s t-test, *P =* 0.013) (**Fig 1D**). This finding suggests that the vaccine-linked chemotherapy administered at early chronic infection prevents cardiac fibrosis caused by *T*. *cruzi* chronic infection. On the other hand, we observed a significantly lower fibrosis percentage in the vaccine alone group (2.043 ± 0.573) or vaccine-linked chemotherapy treated mice compared with low BNZ alone group (3.148 ± 1.065) (Student´s t-test, *P =* 0.030 and *P =* 0.006, respectively). No differences were found comparing the low BNZ + vaccine group with the vaccine alone group (Student´s t-test, *P =* 0.338). For last, E6020-SE treated mice showed a significant increase in cardiac fibrosis compared to vaccine-linked chemotherapy-treated mice (Student´s t-test, *P =* 0.041). All these data suggest that the reduction of cardiac fibrosis area in our experimental infection model is presumably due to the combination of immunotherapy together with BNZ.

**Fig 3:**
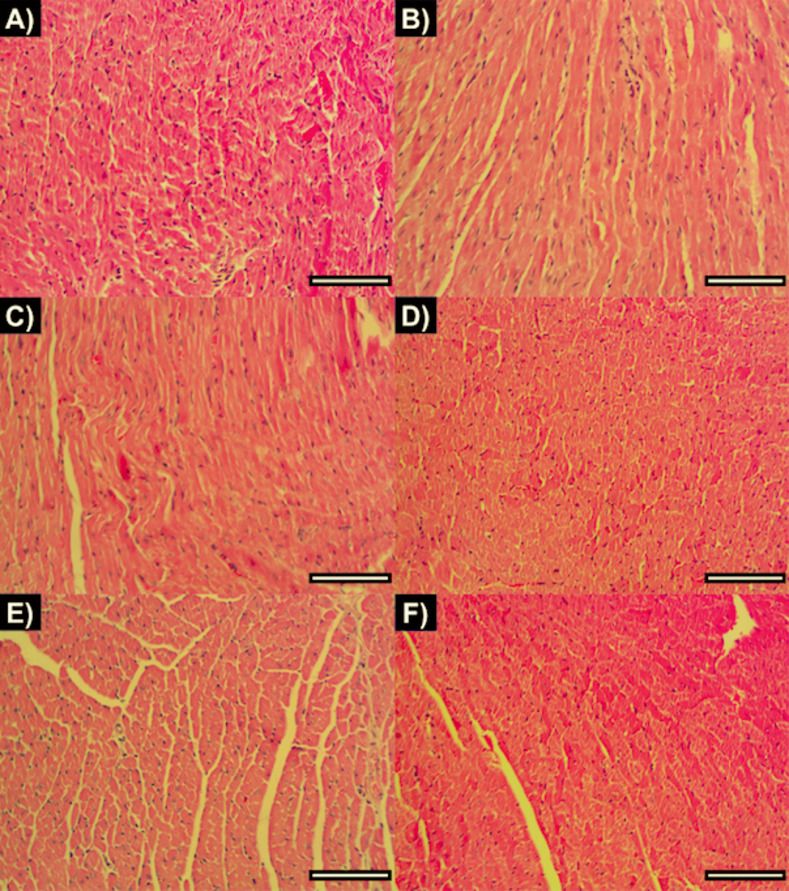
Representative images of H&E stained-cardiac tissue from (**A**) non-infected, (**B**) infected untreated, (**C**) E6020-SE, (**D**) low BNZ, (**E**) vaccine and (**F**) low BNZ plus vaccine experimental groups. Five-nine representative photographs were taken from the myocardial region and were acquired at 20X magnification using a microscope adapted with a digital camera. Cell cytoplasm appears in pink and the nuclei of infiltrating inflammatory cells appear in purple. Scale bar corresponds to 10μm.

We also evaluated the inflammatory infiltrate in heart tissue sections collected from *T*. *cruzi*-infected mice at day 200 p.i. Representative images of H&E stained-cardiac tissue from the different experimental groups are showed in **Fig 3A–3F**. According to **Fig 1E** we observed similar levels of inflammatory cell density between non-infected mice (9,856 ± 1,096) and infected untreated mice (9,544 ± 2,227). Hence, our findings suggest that infected mice at late chronic infection (200 days p.i.) have basal levels of inflammatory infiltrating cells. Furthermore, there were no differences comparing inflammatory cell density from infected untreated mice and the low dose of BNZ + vaccine (8687 ± 1079), vaccine alone (10304 ± 1761), low BNZ alone (9965 ± 1788), or E6020-SE alone (10704 ± 1643) groups (One-way ANOVA, *P* = 0.277) (**Fig 1E**).

### Immunotherapy with low BNZ plus vaccine primes a cytotoxic profile in CD8^+^ and CD4^+^ T antigen-specific cells

**Fig 4:**
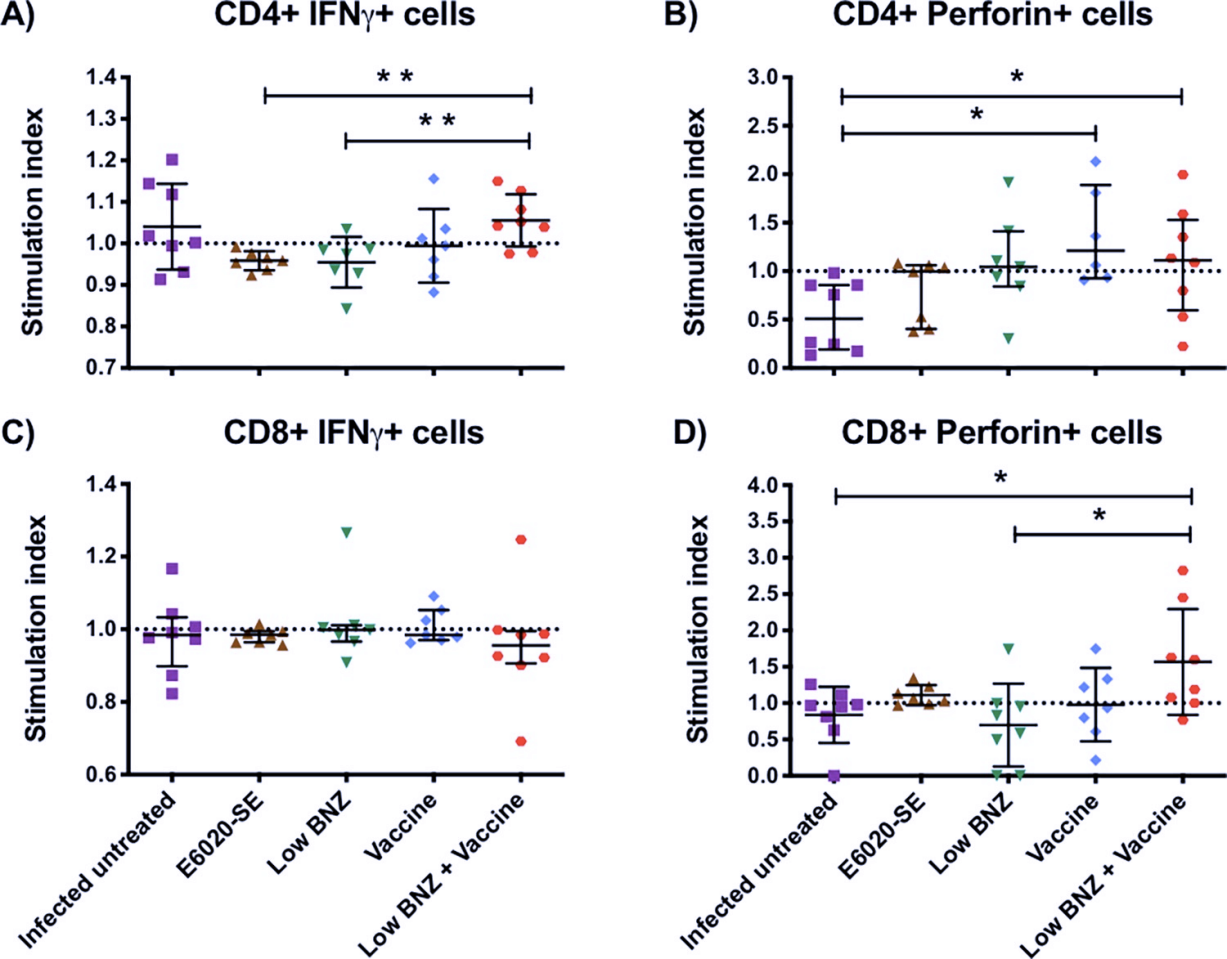
Effect of vaccine-linked chemotherapy on antigen-specific CD4^+^ and CD8^+^ T cells functional profile. Mononuclear cells isolated from mice at 200 days p.i were co-cultivated with macrophages stimulated *in vitro* with TSA-1-C4+Tc24-C4 (25 μg/mL) for 20 h. Data were analyzed using FlowJo X software. Stimulation index of (**A**) antigen-specific IFNγ-producing CD4^+^ cells, (**B**) perforin-producing CD4^+^ cells, (**C**) IFNγ-producing CD8^+^ cells and (**D**) perforin-producing CD8^+^ cells are presented. Each point represents an individual mouse, horizontal lines denote means ± SD or median ± interquartile ranges values according to the normality of the data. Data were analyzed using Student´s t-test or Mann-Whitney U-test. Significance is indicated as follows *, *P*≤0.05; **, *P*≤0.01.

We evaluated the frequency of antigen-specific CD4^+^ and CD8^+^ T cells producing IFNγ (inflammatory profile) and perforin (cytotoxic profile) in mice during the late chronic infection. Mononuclear cells were isolated and co-cultivated with antigen-specific RAW 264.7 macrophages, as described before. According to **Fig 4A,** we observed that CD4^+^ T cells from the low BNZ + vaccine treated group showed a significantly higher stimulation index of CD4^+^IFNγ^+^ cells (1.056 ± 0.062) compared to E6020-SE (0.958 ± 0.023) or low BNZ (0.954 ± 0.06) treated groups (Student´s t-test, *P =* 0.002 and *P =* 0.007 respectively). On the other hand, mice treated with the vaccine alone (1.367 ± 0.504) or the combination low BNZ + vaccine (1.089 ± 0.572) had the highest stimulation index of antigen-specific CD4^+^Perforin^+^ T cells (**Fig 4B**) compared with the infected untreated mice (0.532 ± 0.359) (Mann-Whitney U test, *P =* 0.002 and *P =* 0.049 respectively). No differences were found comparing the vaccine alone group with the low BNZ + vaccine group for inflammatory (Student´s t-test, *P =* 0.143) or cytotoxic (Mann-Whitney U test, *P =* 0.491) profile (**Fig 4A and 4B**), suggesting that there is no benefit of giving BNZ low dose to improve the production of IFNγ or perforin by TSA-1-C4+Tc24-C4 antigen-specific CD4^+^ T cells.

For antigen-specific CD8^+^ T cells, we observed a cytotoxic immuno-phenotype profile characterized by higher perforin-producing CD8^+^ T cells in low BNZ + vaccine-treated mice (1.567 ± 0.728) compared to the infected untreated group (0.838 ± 0.386) or the low BNZ treated group (0.699 ± 0.570) (Student´s t-test, *P =* 0.025, and *P =* 0.018 respectively) (**Fig 4D**). In fact, on average, the perforin production in the vaccine-linked chemotherapy-treated mice was approximately double compared to the infected untreated control group. No differences were found by Kruskal-Wallis (P = 0.702) when we evaluate the production of IFNγ by CD8^+^ T cells (**Fig 4C**). Similarly, no differences were found comparing the vaccine alone group with the low BNZ + vaccine group for inflammatory or cytotoxic CD8+ t cells (Student´s t-test, *P =* 0.097) profile (**Fig 4C and 4D**), suggesting that there is no benefit of giving BNZ low dose to improve the production of IFNγ or perforin by TSA-1-C4+Tc24-C4 antigen-specific CD8^+^ T cells.

### Treatment given during early chronic infection induced a long-lasting *T*. *cruzi*-immunity

We evaluated the memory T cell profile induced by the vaccine-linked chemotherapy, using markers of central (T_CM_) (CD44^+^CD62L^+^) and effector (T_EM_) (CD44^+^CD62L^-^) T cell memory subpopulation in CD4^+^ and CD8^+^ T cells, as described above.

**Fig 5:**
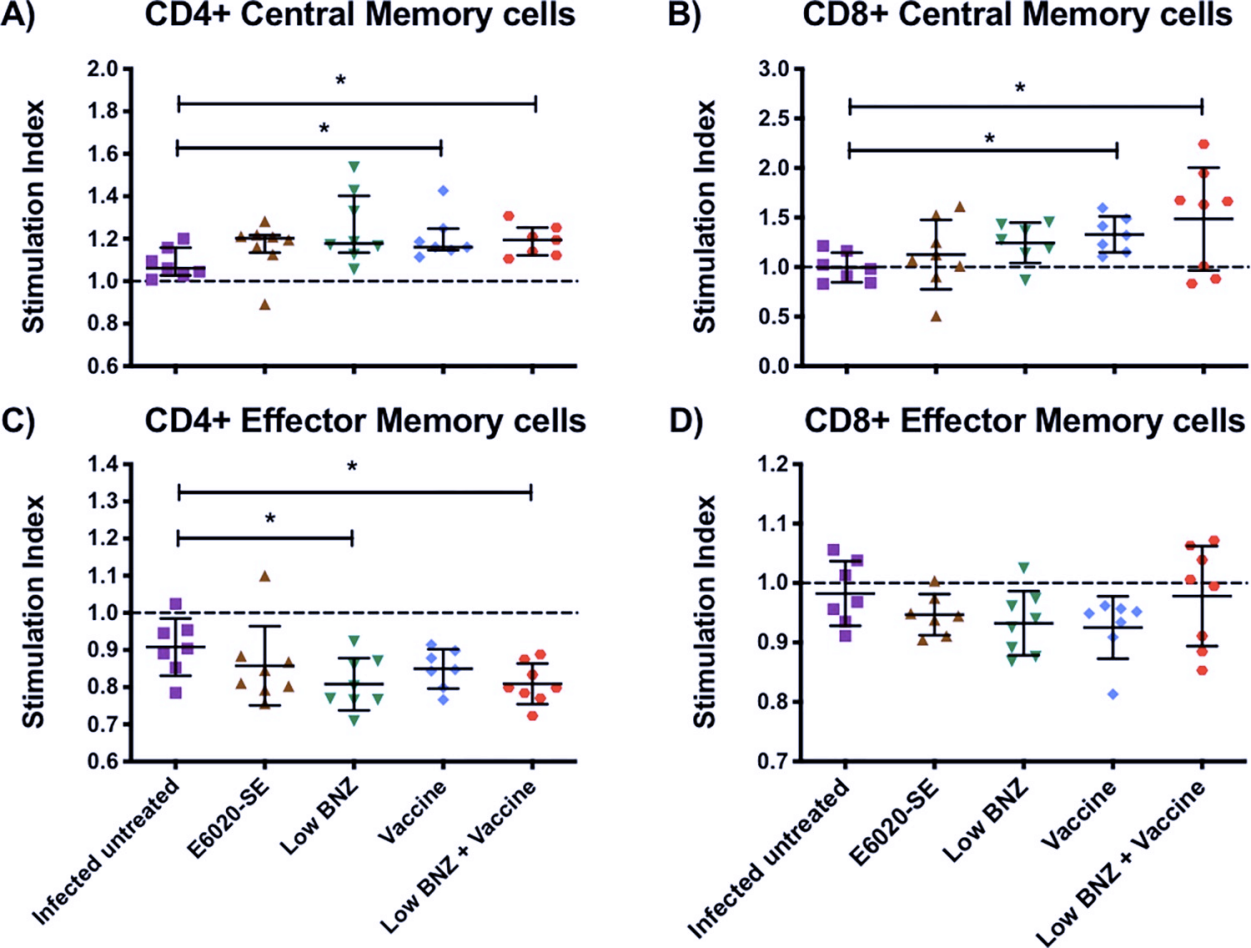
Effect of vaccine-linked chemotherapy on antigen-specific CD4^+^ and CD8^+^ T cells memory response. Mononuclear cells isolated from mice at 200 days p.i. were co-cultivated with macrophages stimulated *in vitro* with TSA-1-C4+Tc24-C4 (25 μg/mL) for 96 h. Data were analysed using FlowJo X software. Stimulation index of antigen-specific (**A**) CD4^+^ Central Memory, (**B**) CD8^+^ Central Memory, (**C**) CD4^+^ Effector Memory and (**D**) CD8^+^ Effector Memory T cells are presented. Each point represents an individual mouse, horizontal lines denote mean ± SD or median ± interquartile ranges values according to the normality of the data. Data were analysed using Student´s t-test or Mann-Whitney U-test. Significance is indicated as follows *, *P*≤0.05.

As observed in **Fig 5A**, we found a significantly higher stimulation index of antigen-specific CD4^+^ T_CM_ sub-population in the low BNZ + vaccine treated mice (1.19 ± 0.073) and vaccine alone groups (1.206 ± 0.105) compared to infected untreated mice (1.085 ± 0.07) (Mann-Whitney U test, *P =* 0.026 and *P =* 0.023 respectively). Similarly, a significantly higher stimulation index of CD8^+^ T_CM_ sub-population (**Fig 5B**) was observed in low BNZ + vaccine treated mice (1.485 ± 0.519) and vaccine alone groups (1.33 ± 0.18) compared to infected untreated mice (0.995 ± 0.149) (Student´s t-test, *P =* 0.032 and *P =* 0.002 respectively). We did not observe significant differences comparing the vaccine alone group with the low BNZ + vaccine group for both, CD4^+^ or CD8^+^ T_CM_ cells (Mann-Whitney U test, *P =* 0.972 and *P =* 0.466 respectively) (**Fig 5A and 5B**). This data suggests that the vaccine alone elicited TSA-1-C4+Tc24-C4 antigen-specific central memory response during the late chronic phase of *T*. *cruzi*-infection in BALB/c mice. Accordingly, the addition of BNZ low dose had no effect on the long-lasting immune response specific to the antigens in the vaccine formulation. Otherwise, we observed that all experimental groups had a mean stimulation index ≤ 1 for CD4^+^ and CD8^+^ T_EM_ cells populations (**Fig 5C and 5D**). This data indicates that, the recombinant protein vaccine does not stimulate the effector memory population during the late chronic phase of the infection (200 days p.i.).

## Discussion

One of the greatest challenges in chronic CD is the development of therapies that improve prognosis and, even, reverse cardiac injury. Our research partnership is developing a therapeutic vaccine against CD that seeks to prevent or delay the onset of CCC in infected patients. Previous studies by our group have evidenced the feasibility of DNA vaccines formulated with TSA-1 and Tc24 *T*. *cruzi*-antigens in mice and dogs [46,58,59]. This DNA-bivalent vaccine was associated with a CD8^+^ T cell activity, IFNγ production, Th1 immune response. Since DNA vaccines historically have not yet progressed successfully to emergency use authorization or full licensure for use in humans, our studies have focused on recombinant protein antigens [60]. Hence, we embarked on studies to evaluate vaccines formulated with TSA-1 or Tc24 recombinant proteins in conjunction with TLR-4 agonist adjuvants [40,55]. These previous studies have concluded that either Th1 or balanced Th1/Th2 immune responses are associated with reductions in parasite burdens, fibrosis, and inflammatory infiltrate linking it to confer protection in experimental models of acute *T*. *cruzi* infection [23,41,42].

Specific genetic mutations have been made for both recombinant proteins, to facilitate production and increase stability, while maintaining immunogenicity in mice; the proteins resulting were named as Tc24-C4 [38,39] and TSA-1-C4. These designations reflect the modification of cysteines to prevent protein aggregation from intermolecular disulphide bond formation. The bivalent vaccine, therefore, is comprised of two recombinant *T*. *cruzi* antigens, Tc24-C4 and TSA-1-C4, which demonstrated immunogenicity in non-human primate trials study [45]. Moreover, recent studies have aimed to evaluate BNZ in reduced dosing regimens to decrease adverse side effects [47,49]; hence, these studies, support the evaluation of combination therapies based on a reduced low dose of BNZ linked to our bivalent recombinant protein vaccine. However, effective therapeutic treatment for CD must prove its effectiveness in preventing or delaying the onset of CCC [24]. Thereby, it is critical to evaluate therapeutic treatments in pre-clinical models during the chronic phase of *T*. *cruzi*-infection. Here, we evaluated the therapeutic efficacy of a vaccine candidate formulated with TSA-1-C4 + Tc24-C4 recombinant antigens combined with a 4-fold reduction in the amount and dosage regimen of BNZ treatment given during the early chronic phase of *T*. *cruzi*-infection in a murine model and followed until the late chronic phase of the disease.

In terms of protective effect, diminished parasitemia and cardiac parasite burdens are related to the therapeutic efficacy of vaccines in murine models of *T*. *cruzi* acute infection [22,41,42,44,55]. On the other hand, *T*. *cruzi* parasites in blood and cardiac tissue decrease with time during *T*. *cruzi* chronic infection becoming undetectable in blood and restricted in tissues, where they are not always demonstrable, even by using highly sensitive amplification techniques such as qPCR assay [61]. However, novel techniques using a bioluminescence imaging system have allowed measuring the parasite burden *in vivo* during *T*. *cruzi*-chronic infection demonstrating that the *T*. *cruzi* presence in the heart is spatially dynamic ([62]. This finding suggests that parasitemia, as well as cardiac parasite burden, are not robust indicators to evaluate therapeutic efficacy during *T*. *cruzi* chronic infection in murine models. Therefore, the absence of detectable parasites in the heart at a point of infection does not necessarily exclude the ongoing infection itself, as the parasite could be restricted to other organs, such as in the gastrointestinal tract (62). This could explain our results, as we detected low levels of parasite burden in the heart. Further studies need to be performed to understand the mechanism that allows the establishment of cardiac disease with irregular levels of parasites.

Beyond parasite reduction, there are findings stressing the role of reducing both cardiac fibrosis and inflammation in patients, non-human primates, and mice [63,64]. In fact, the use of non-invasive methods to measure fibrosis has allowed distinguishing potentially useful biomarkers of cardiac fibrosis, such as TGF-β, connective tissue growth factor, and platelet-derived growth factor-D [56]. In the current study, we showed that infected untreated mice in chronic infection exhibited a more severe cardiac fibrosis compared to the non-infected control group, as expected. Also, we observed that the vaccine-linked chemotherapy-treated group had on average 50% of the cardiac fibrosis area compared to the infected untreated group. Thus, we demonstrated that the vaccine-linked chemotherapy given during the early chronic phase of *T*. *cruzi* infection can prevent cardiac fibrosis in our mouse model. This finding support previous studies in mice during acute infection by *T*. *cruzi* showing that Tc24, Tc24-C4 immunizations or the vaccine-linked chemotherapy can reduce parasite burden and cardiac fibrosis [23,44]. In this experimental infection model, the administration of the low dose of BNZ alone (25mg/kg for 7 days) was unable to prevent cardiac fibrosis evaluated at 200 days p.i. These results coincide with previous studies, showing that BNZ therapy (100mg/kg for 20 days) prevents the development of cardiac fibrosis in the murine model when treatment is administrated in the acute phase, however, the drug fails when is administrated during the chronic phase of infection [12].

As part of the study, we intended to evaluate protection against cardiac inflammation conferred by our vaccine-linked chemotherapy. However, we observed similar levels of inflammatory infiltrate in cardiac tissue from infected untreated and non-infected mice, suggesting that at day 200 p.i. the inflammatory infiltrate in cardiac tissue reaches basal levels, confirming what has been previously described by Hoffman et al [56].

Previously, it has been demonstrated that Th17 cytokines family plays a critical role in host survival by regulating inflammation and immunopathology during *T*. *cruzi* infection, suggesting that IL-17, as well as other regulatory cytokines, could have protective roles during adaptive immunity and therefore, regulate inflammation in tissues, such as heart [44,65,66]. Hence, further studies, are needed to evaluate Th17 and regulatory cytokine profile induced by the recombinant TSA-1.C4 plus Tc24.C4 antigen combination.

The CD8^+^ cytotoxic T cells activation is essential to achieve immunity against *T*. *cruzi* parasite [67,68]. We showed that the vaccine-linked chemotherapy-treated group had on average double production of perforin by antigen-specific CD8^+^ T cells compared to infected untreated mice. Our results pointed out that the immunotherapy with TSA-1-C4+Tc24-C4 recombinant antigens is able to prime a cytotoxic profile (CD8^+^Perf^+^) during the late chronic phase of *T*. *cruzi*-infection. Also, we observed that the vaccine alone group, as well as the vaccine-linked chemotherapy, elicited an antigen-specific CD4^+^ CTL sub-population, these cells can secrete cytotoxic granules that directly kill infected cells in an MHC-class-II-restricted context. Previous studies have described CD4^+^ CTL sub-population in both, human and murine models [69–71]. Currently, the mechanism used by CD4^+^ CTL is unclear, however; this sub-population can exhibit functions complementary to CD8^+^ CTLs [72]. More studies are needed to elucidate the role of CD4^+^ CTL in *T*. *cruzi* infection.

On the other hand, we did not observe significant differences in either antigen-specific CD4^+^IFNγ^+^ or CD8^+^IFNγ^+^-producing T cells in the vaccine-linked chemotherapy-treated mice compared with the infected untreated group. Both phenotypes are characteristic of the Th1 immune response that is known to confer protection against *T*. *cruzi* acute infection. Although a proinflammatory profile mediated by IFNγ producing CD4^+^ and CD8^+^ T cells is desirable, in this experimental chronic infection model we observed that the vaccine-linked immunotherapy drives a cytotoxic profile characterized by perforin producing CD4^+^ and CD8^+^ T cells at 200 days post-infection.

A challenge in the development of an effective vaccine against *T*. *cruzi* is the induction of long-lived memory cells, which confers long-term protection. In our study, we were able to recall antigen-specific CD4^+^ and CD8^+^ T_CM_ sub-populations at 200 days p.i. The central memory T cells are distinguished for having a proliferative response followed by antigenic stimulation that lives longer than effector memory cells. Bixby and Tarleton have previously reported this sub-population in CD8^+^ T cells during *T*. *cruzi*-infection in mice showing distinctive features called T_CM_ cells [35]. Similarly, T_EM_ sub-population represents a type of terminally differentiated cells that produce IFNγ and IL-4 or contain pre-stored perforin [33]. In this study, we observed a low stimulation index of either CD4^+^ or CD8^+^ T_EM_ sub-populations regardless of treatment from infected mice at 200 days p.i. However, the use of the recombinant protein vaccine alone or the vaccine-linked chemotherapy recalls a strong central memory response mediated by antigen-specific T cells at 200 days p.i. showing that the vaccine formulation is responsible of the long-lasting antigen-specific memory immune response elucidated.

The current study presents new findings for the development of combined therapies against *T*. *cruzi*, contributing to the research of CD. This is the first report showing the effects induced by the bivalent recombinant protein vaccine (TSA-1-C4+Tc24-C4+E6020-SE) with a 4-fold reduction dose of BNZ (25mg/kg/7 days), given during early chronic phase and followed until late chronic phase of CD (200 days p.i.). In addition, we demonstrate for first time that, the vaccine-linked chemotherapy prevents the development of cardiac fibrosis and exhibit a cytotoxic profile mediated by CD4^+^ and CD8^+^ T cells, as well as a long-term immune response characterized by T_CM_ cells in BALB/c murine model. We also present novelties in the methodology section, such as the use of RAW 264.7 macrophages stimulated with recombinant proteins to perform them as APCs and promote the immunological synapses.

There are some limitations in this study. We did not measure clinical parameters to assess heart function. Therefore, we were not able to correlate the reduced fibrosis with improved clinical cardiac output in our chronically infected mice. We are aware of the prolonged time needed to evaluate the vaccine-linked chemotherapy efficacy in the chronic phase limiting the evaluation of the vaccine immunogenicity at different time points; therefore, future studies should include extra control groups to evaluate protection parameters and immune responses at different time points of the infection. Besides, we observed variability in our data, probably due to the sample size, however, the reduced number of mice per group was due to the high mortality rates observed during the acute phase of the infection (43% of mortality). Nevertheless, this demonstrates that only mice that survived the lethal infection and remain in asymptomatic chronic phase received the therapeutic intervention. In addition, the BALB/c model of experimental infection used here seems to have an intrinsic resistance to *T*. *cruzi* acute infection allowing them to progress until the late chronic phase, which is characterized by lower levels of parasite burden and inflammation but higher percentages of cardiac fibrosis in infected untreated mice. However, our BALB/c model may be representative of a majority proportion of the *T*. *cruzi* infected human population that can control parasite burden and inflammation, remaining in the asymptomatic chronic phase of CD for life.

## Conclusion

We demonstrate that treatment with a low dose BNZ and a vaccine immunotherapy during early chronic infection protects mice against cardiac fibrosis progression and induces a long-lasting *T*. *cruzi*-immunity that persists for at least 18 weeks post-treatment. Although the recombinant protein vaccine alone is enough to stimulate the immune memory response, the administration of the low dose of BNZ is necessary for reduction of cardiac fibrosis. This study supports the use of a vaccine-linked chemotherapy approach given during early chronic infection, however; additional studies in other preclinical models that develop CCC and with more characteristics of human disease, such as non-human primates, will be necessary before the combination of a vaccine-linked to BNZ can be moved into clinical trials.

## Supporting information

S1 Table Parasitemia measurement. A total of 70 BALB/c mice were infected with 500 trypomastigotes of *T*. *cruzi* (H1 strain) by intraperitoneal injection. A total of 4 mice were used as non-infected control group and received only saline solution. Parasitemia was measured in Neubauer chamber by examination of fresh blood collected from the mouse tail at day 27 post-infection. All infected mice were positive for *T*. *cruzi*, while neither mouse from the non-infected control group was reported as positive. **SD**, standard deviation; **CI**, confidence interval.
(TIF)

S1 Fig Protein integrity assessment. SDS-PAGE analysis at 12% of acrylamide/bis-acrylamide and stain with PageBlue Protein Staining Solution (ThermoFisher Scientific). Molecular weight marker (Spectra Multicolor Broad Range Protein Ladder from ThermoFisher Scientific) and recombinant proteins TSA-1-C4 (65 kDA) and Tc24-C4 (24 kDa) are presented.
(TIF)

S2 Fig Flow-cytometry gating strategy for IFNγ and perforin production. The dot-plots show the mononuclear cells gating based on (**A**) forward-scatter (FSC) and side-scatter (SSC) properties, (**B**) doublets exclusion, (**C**) identification of CD3^+^ positive cells, (**D**) phenotype of CD4^+^ and CD8^+^ cells and (**E**) IFNγ and perforin expression. Gates were established using the non-stained and Frequency Minus One (FMO) controls.
(TIF)

S3 Fig Flow-cytometry gating strategy for central and effector memory response. The dot-plots show the mononuclear cells gating based on (**A**) forward-scatter (FSC) and side-scatter (SSC) properties, (**B**) doublets exclusion, (**C**) identification of CD3 positive cells, (**D**) phenotype of CD4^+^ and CD8^+^ cells and (**E**) central memory and effector memory profile defined by (CD44^+^CD62L^+^) and (CD44^+^CD62L^-^) expression respectively. Gates were established using the non-stained and Frequency Minus One (FMO) controls.
(TIF)
