# Supplementary figures and images for: Vaccine-linked chemotherapy with a low dose of benznidazole plus a bivalent recombinant protein vaccine prevents the development of cardiac fibrosis caused by Trypanosoma cruzi in chronically-infected BALB/c mice
Source: PLoS Negl Trop Dis. 2022 Sep 12;16(9):e0010258. doi: 10.1371/journal.pntd.0010258 (PMC9499242; doi:10.1371/journal.pntd.0010258)

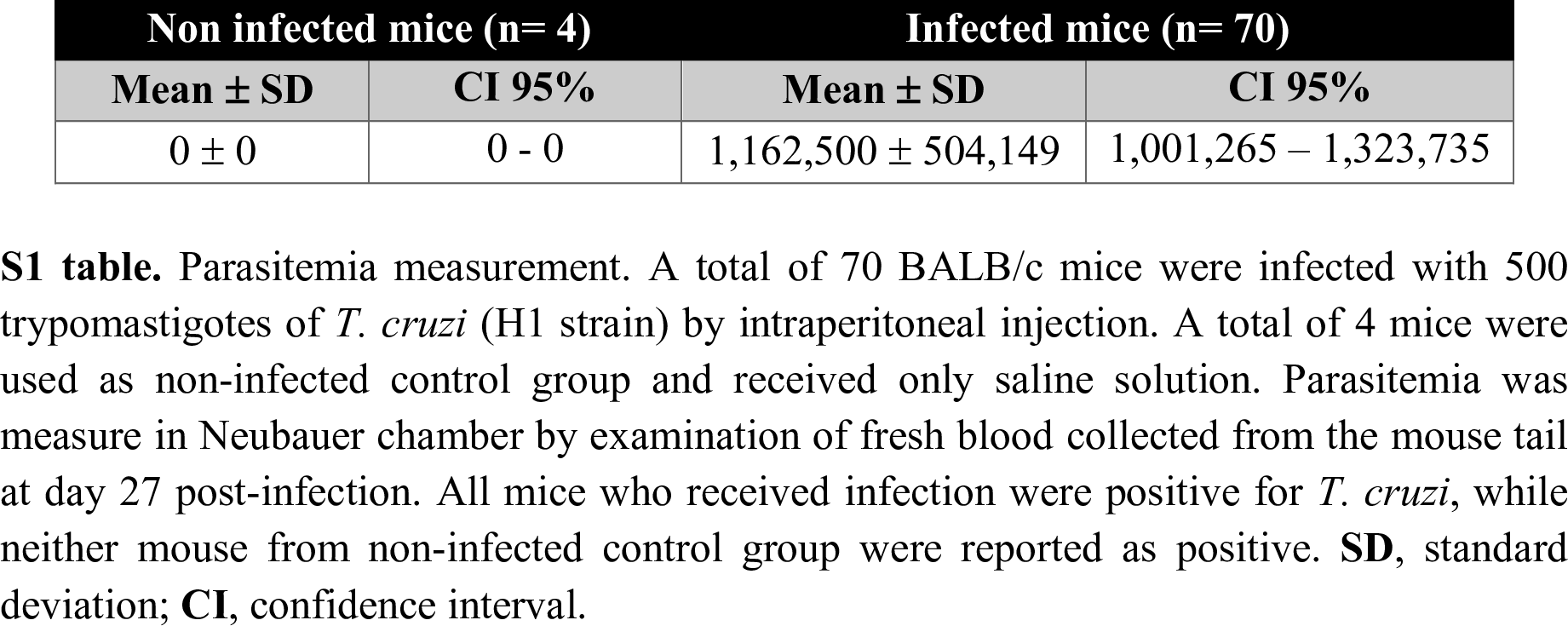

Supplement: S1 Table — A total of 70 BALB/c mice were infected with 500 trypomastigotes of T. cruzi (H1 strain) by intraperitoneal injection. A total of 4 mice were used as non-infected control group and received only saline solution. Parasitemia was measured in Neubauer chamber by examination of fresh blood collected from the mouse tail at day 27 post-infection. All infected mice were positive for T. cruzi, while neither mouse from the non-infected control group was reported as positive. SD, standard deviation; CI, confidence interval. (TIF) [file pntd.0010258.s001.tif]

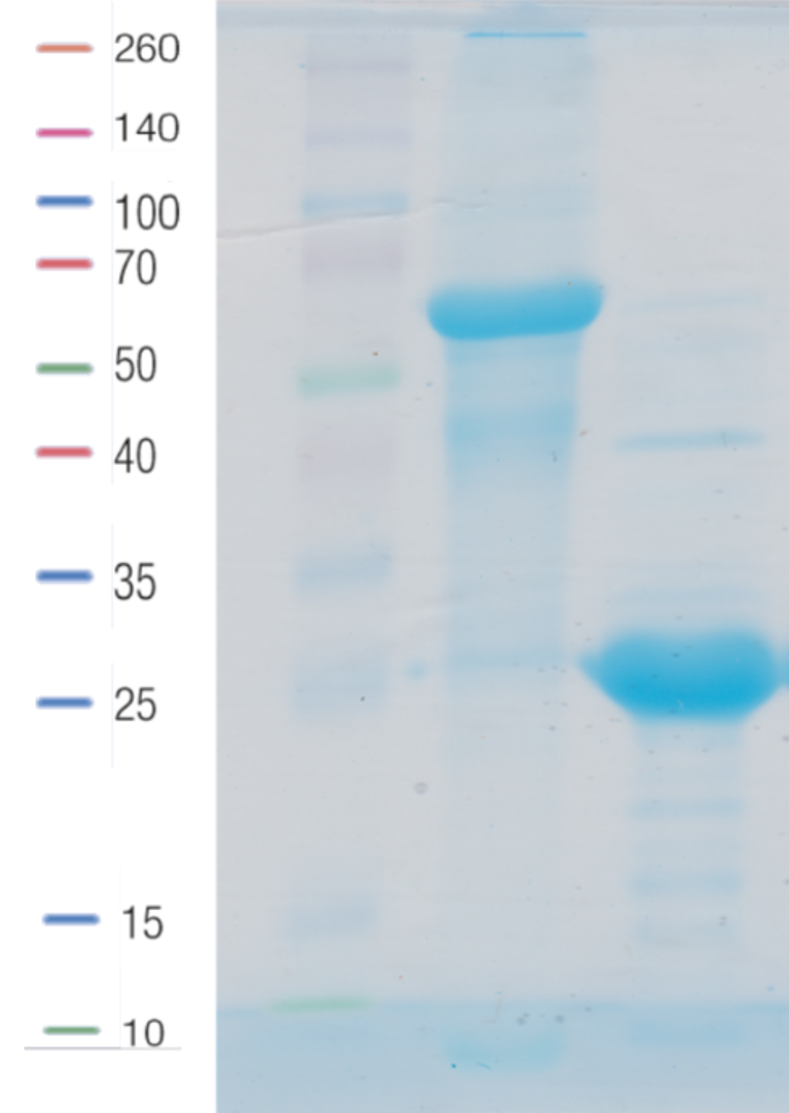

Supplement: S1 Fig — SDS-PAGE analysis at 12% of acrylamide/bis-acrylamide and stain with PageBlue Protein Staining Solution (ThermoFisher Scientific). Molecular weight marker (Spectra Multicolor Broad Range Protein Ladder from ThermoFisher Scientific) and recombinant proteins TSA-1-C4 (65 kDA) and Tc24-C4 (24 kDa) are presented. (TIF) [file pntd.0010258.s002.tif]

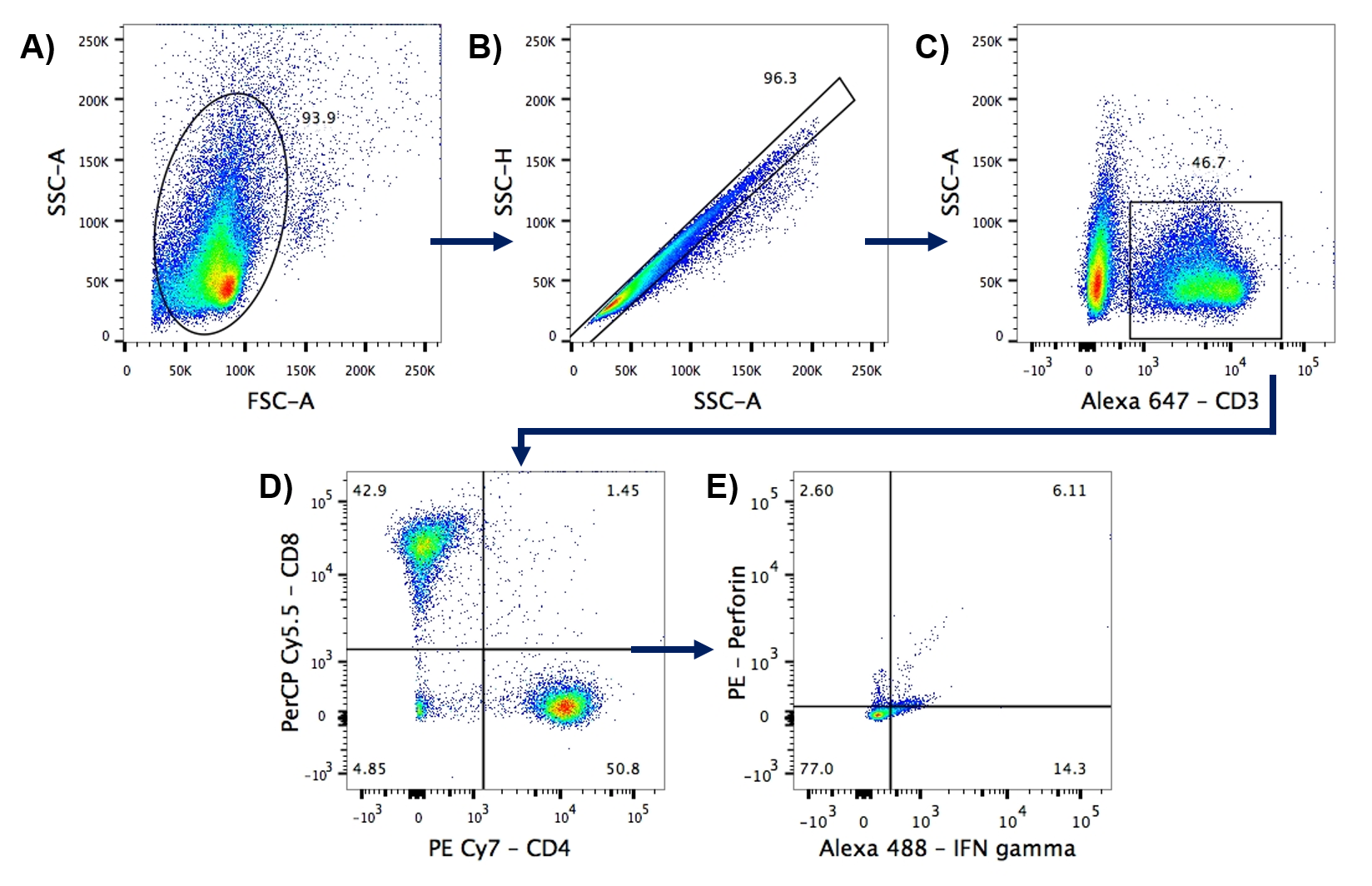

Supplement: S2 Fig — The dot-plots show the mononuclear cells gating based on (A) forward-scatter (FSC) and side-scatter (SSC) properties, (B) doublets exclusion, (C) identification of CD3+ positive cells, (D) phenotype of CD4+ and CD8+ cells and (E) IFNγ and perforin expression. Gates were established using the non-stained and Frequency Minus One (FMO) controls. (TIF) [file pntd.0010258.s003.tif]

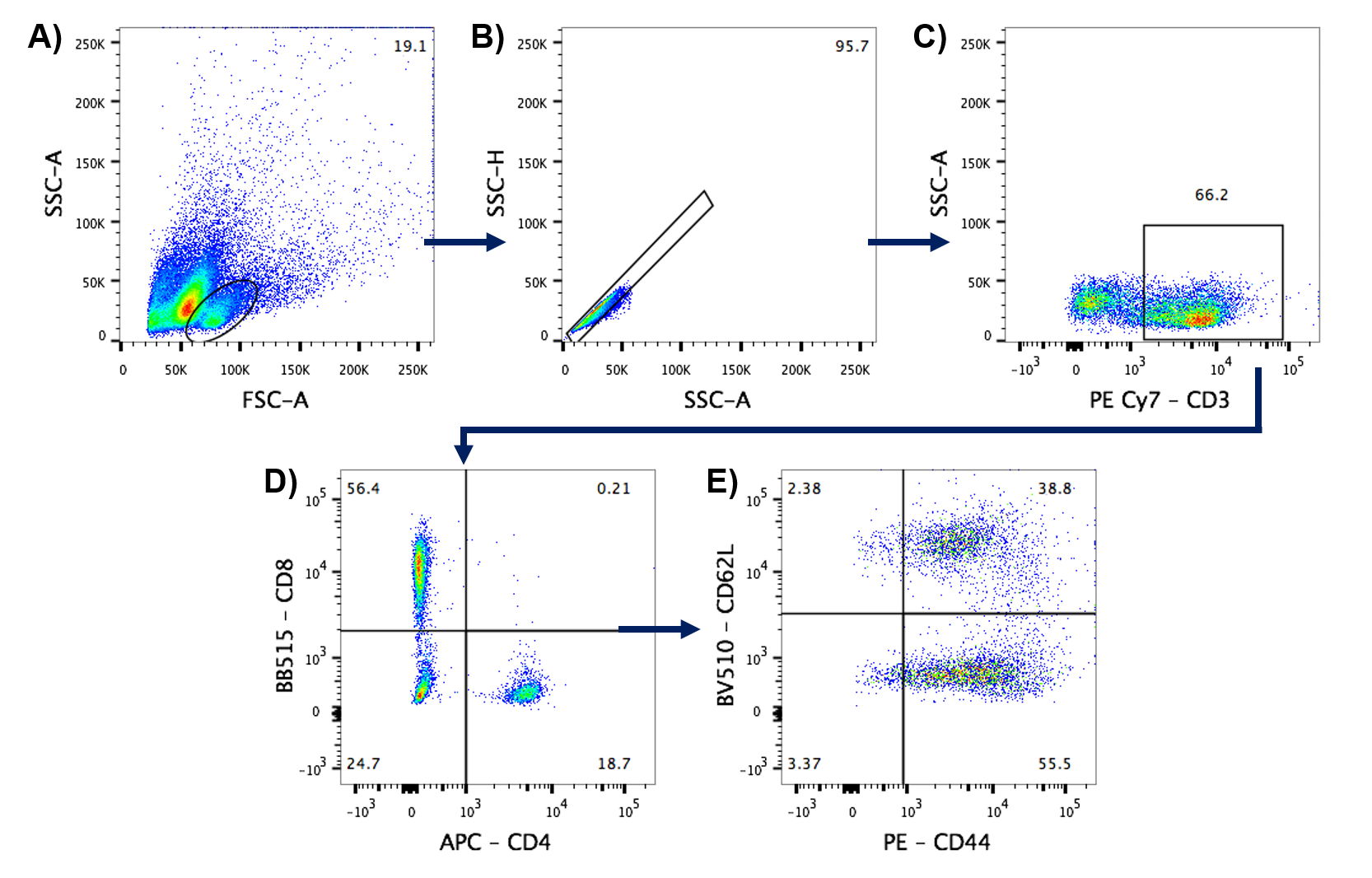

Supplement: S3 Fig — The dot-plots show the mononuclear cells gating based on (A) forward-scatter (FSC) and side-scatter (SSC) properties, (B) doublets exclusion, (C) identification of CD3 positive cells, (D) phenotype of CD4+ and CD8+ cells and (E) central memory and effector memory profile defined by (CD44+CD62L+) and (CD44+CD62L-) expression respectively. Gates were established using the non-stained and Frequency Minus One (FMO) controls. (TIF) [file pntd.0010258.s004.tif]
